# Differences of Sleep Disorders Between Vestibular Migraine and Benign Paroxysmal Positional Vertigo

**DOI:** 10.3389/fpsyt.2021.726038

**Published:** 2021-11-18

**Authors:** Hui Xue, Baojun Wang, Tianyu Meng, Shijun Zhao, Qingyin Wang, Xin Zhang, Min Kang, Wenping Xiang

**Affiliations:** Department of Neurology, Baotou Central Hospital, Baotou, China

**Keywords:** sleep disorders, vestibular migraine, benign paroxysmal positional vertigo, VM, BPPV

## Abstract

**Introduction:** Sleep disorders can affect the overall health and quality of life of patients. This study was conducted to compare the differences of sleep disorders in vestibular migraine (VM) patients and benign paroxysmal positional vertigo (BPPV) patients.

**Methods:** VM patients, BPPV patients, and healthy controls (HCs) were recruited. Pittsburgh sleep quality index and polysomnography monitoring were used as subjective and objective, respectively, evaluation methods to evaluate the sleep quality of participants in the latest month.

**Results:** Fifty-seven BPPV patients, 48 VM patients, and 42 HCs were included in this study. There were 79.16% VM patients, 54.39% BPPV patients, and 14.28% HCs with sleep disorders. The difference in the incidence rate of sleep disorders was significant between VM patients and BPPV patients (*p* = 0.008) and significantly higher in both the VM group (*p* < 0.00001) and BPPV group (*p* = 0.00004) than in the HC groups (14.28%). Compared with BPPV patients, the VM patients had the significantly lower sleep efficiency (*p* < 0.001) and N3 (*p* < 0.001) and the significantly higher time of wake-up after sleep onset (*p* < 0.001), N1 (*p* < 0.001), and N2 (*p* < 0.001). Meanwhile, the VM patients had significantly higher incidence rates of severe obstructive sleep apnea hypoventilation syndrome (*p* = 0.001) and periodic leg movement in sleep (*p* = 0.016).

**Conclusion:** The incidence rate of sleep disorders was significantly higher in both VM and BPPV patients than in the HC groups. To improve the curative effects, clinicians should pay more attention to the comorbidity of sleep disorders in treating VM and BPPV.

## Introduction

Sleep disorders are a group of conditions that can affect the quality of sleep. Sleep disorders and dizziness/vertigo are common clinical diseases with a high incidence rate. A previous study reported that there was a close relationship between sleep disorders and dizziness/vertigo: (i) sleep disorders will affect the therapeutic effect of drugs and quality of life of patients with dizziness/vertigo (1), and (ii) dizziness/vertigo attacks will interfere with the patient's sleep (2). Sowerby et al. found that sleep apnea and daytime sleepiness were associated with idiopathic dizziness, and they suggested that sleep disorders might have diagnostic value and prognostic prediction value for vestibular diseases (3). Although its etiology is still needed to be further explored, there may be a certain relationship between sleep disorders and dizziness/vertigo.

Vestibular migraine (VM) is a headache disorder with symptoms such as vertigo or dizziness (vestibular symptoms) and is very sensitive to light/sound, headache, and nausea (migraine symptoms) (4). The pathogenesis of VM is still not clear. According to the pathogenesis of migraine, many researchers believe that both peripheral and central vestibular systems have an important role in the onset of VM (5). Benign paroxysmal positional vertigo (BPPV) is viewed as the most common form of peripheral vertigo. In recent decades, many theories have been developed to explain the pathogenesis of BPPV, but it is generally accepted that the abnormal blood supply of the inner ear may be the cause of most primary BPPV (6). No matter what the pathogenesis of BPPV is, it is clear that BPPV also belongs to peripheral vestibular disease.

Both VM and BPPV are viewed as paroxysmal vertigo in clinical practice, but the pathogenesis of these two diseases is different (7, 8). Meanwhile, the time and characteristics of vertigo attacks are also different between VM and BPPV (9, 10). Generally speaking, VM and BPPV are the most common dizziness/vertigo diseases; thus, it is of clinical significance to compare the sleep status of these two diseases. In this study, we recruited patients with VM and patients with BPPV to observe their sleep status. We used both subjective and objective sleep quality evaluation methods to assess their characteristics of sleep disorders, sleep microstructure, and sleep-related diseases.

## Methodology

### Participants' Recruitment

VM patients and BPPV patients were recruited from the vertigo center of our hospital from December 1, 2016, to May 31, 2017. Meanwhile, healthy controls (HCs) were recruited from the Medical Examination Center of our hospital. Patients were excluded if they: (i) had a clear history of central vestibular injury (such as stroke, multiple sclerosis, neuromyelitis optica, etc.), (ii) were drug and alcohol abusers, (iii) received sedative drugs (benzodiazepines, barbiturates, and other drugs that might affect the central system) 1 week before enrollment, (iv) had anxiety/depression symptoms or a history of mental illness, (v) had a diagnosed insomnia and were unable to complete sleep monitoring, (vi) had severe medical diseases or specific diseases affecting the quality of sleep, (vii) were unable to cooperate with the assessment or inspection, and (viii) had trauma and recent operation (within 3 months).

### Subjective Sleep Quality Evaluation Method

Pittsburgh Sleep Quality Index (PSQI) was used to evaluate the sleep quality of participants in the latest month. It consists of nine self-rated items and five physician-rated items. There are 18 sub-items in the 9 self-rated items, which can be combined into 7 factors: sleep quality, sleep latency (SL), sleep time (ST), sleep efficiency (SE), sleep disorder-related factors, hypnotics, and daytime function. Each factor is scored according to the grade (ranged from 0 to 3), and the total score of each factor was the total score of PSQI. The five physician-rated items do not participate in the scoring; thus, these items are not assessed here. The total score of PSQI ranges from 0 to 21. The higher the total score, the worse the sleep quality. The sleep quality will be considered as good (PSQI score ≤ 5), moderate (5 < PSQI score ≤ 7), and poor (PSQI score > 7) according to the total score of PSQI (11). The PSQI assessment was conducted at the beginning of recruitment.

### Objective Sleep Quality Evaluation Method

The polysomnography was performed using a polysomnography monitor (Nicolet v32, Natus Medical Incorporated, Pleasanton, CA, USA). BPPV patients were evaluated in the recovery period of complete vertigo remission, and VM patients were evaluated in the intermission period of vertigo attacks. All participants were evaluated at the sleep electroencephalogram center monitoring room of our hospital, and the sleep was not affected by the outside world. The monitoring time was from 9:00 p.m. to 6:00 a.m., and the bed rest time was more than 7 h. Coffee, coke, tea, painkillers, and cold medicines were prohibited from the day before the examination. The monitoring indexes included electroencephalogram, electrooculogram, thoracic and abdominal respiratory movement, transcutaneous oxygen saturation, and heart rate.

### Physiological Parameters of Sleep and Its Definitions

The total ST is defined as the time from the beginning of sleep to the end of sleep (not including the time of waking up during the experiment). The total bedtime (BT) is defined as the time from turning off the light on the first night to waking up the next morning. The SE is defined as the proportion of total ST to total TBT. The number of wake-ups during the experiment is recorded. The time of wake-up after sleep onset (WASO) is defined as all wake-up time from sleep onset to the last wake-up. The SL is defined as the time from turning off the light to non-rapid eye movement (REM). The REM-SL is defined as the time from the sleep onset to the first REM.

The Apnea–Hypopnea Index (AHI) and Periodic Leg Movement in Sleep (PLMS) are also recorded. Normally, both the numbers of AHI and PLMS are less than five times/hour in a normal population. The obstructive sleep apnea hypoventilation syndrome (OSAHS) is diagnosed with the number of AHI ≥ 5 and snoring, daytime sleepiness, sleep apnea, and other clinical symptoms. The OSAHS is considered as mild (5 ≤ AHI < 15), moderate (15 ≤ AHI < 30), and severe (AHI ≥ 30). The PLMS mainly appears in shallow sleep periods, especially in the N1 and N2 stages, which is characterized by periodic episodes of highly and repetitive stereotypical movements of the lower extremities. The PLMS is diagnosed with the number of limb movements ≥ 4, 20–40-s interval, and 0.5–5-s lasting time.

### Statistically Analysis

All analyses in this study were conducted using SPSS 22.0 software. *P* < 0.05 was set to be significantly different. Continuous variables were expressed as mean ± standard deviation, and counting variables were presented as frequency and percentage (12, 13). The Student's *t*-test, nonparametric Mann–Whitney *U*-test, or one-way analysis of variance was used to analyze the continuous data (14–16). If a significant difference was found in one-way analysis of variance, *post-hoc* Bonferroni correction was used to find out which groups differed significantly (17). The Chi-square test was used to analyze the counting data.

## Results

In total, 57 BPPV patients, 48 VM patients, and 42 HCs were included in this study. There were 22 males and 35 females in the BPPV group, 18 males and 30 females in the VM group, and 18 males and 24 females in the HC group. There was no significant difference in sex ratio among the three groups. The average age was 54.68 (10.81) years in the BPPV group, 55.92 (10.11) years in the VM group, and 56.81 (9.44) years in the HC group. There was no significant difference in average age among the three groups. The detailed information of these included patients is described in Table 1.

**Table 1:** Demographic characters in BPPV, VM patients, and HCs.

**Variables**	**BPPV group**	**VM group**	**HCs group**	***P***
Number	57	48	42	–
Age (years)	54.68 ± 10.81	55.92 ± 10.11	56.81 ± 9.44	0.72^a^
Sex (F/M)	35/22	30/18	24/18	0.86^b^
BMI (kg/m^2^)	25.61 ± 4.58	25.38 ± 5.02	25.72 ± 4.86	0.69^a^
Duration of disease^d^	–	2.31 ± 0.82 years	–	–
Frequency of vertigo	1.21 ± 0.25 time/minute	4.27 ± 1.53 time/month	–	<0.0001^c^
Duration of each vertigo	55.2 + 15.7 second/time	3.68 ± 2.27 hour/time	–	<0.0001^c^
DHI	55.11 ± 17.59	49.40 ± 15.47	–	0.08^c^

a *P-value was obtained from a one-way analysis of variance*.

b *P-value was obtained from Chi-square test*.

c *P-value was obtained from Student's t-test*.

d *Patients in BPPV group were patients with first attack of vertigo*.

*F, female; M, male; BMI, body mass index; BPPV, benign paroxysmal positional vertigo; VM, vestibular migraine; HCs, healthy controls; DHI, dizziness handicap inventory*.

### Subjective Sleep Quality Evaluation

**Figure 1:**
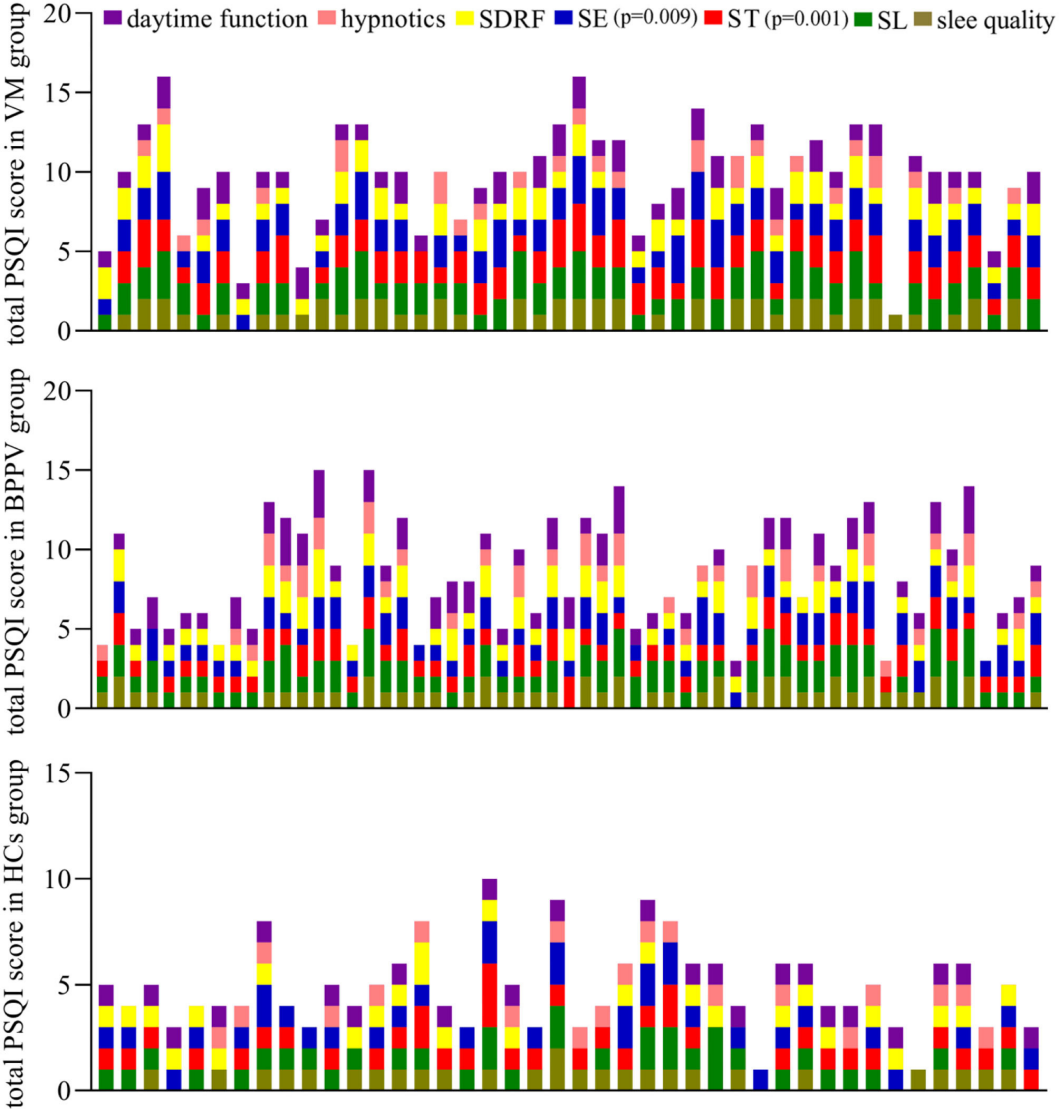
Comparison of PSQI scores in BPPV patients, VM patients, and HCs.

The average total PSQI score was 9.81 ± 3.11 in the VM group, 8.51 ± 3.34 in the BPPV group, and 4.88 ± 2.01 in the HCs group. The VM group had a significantly higher average total PSQI score compared with the BPPV group (*p* = 0.043), which indicated that the VM patients had worse sleep disorders than BPPV patients. Both the VM group (*p* < 0.00001) and the BPPV group (*p* < 0.00001) had a significantly higher average total PSQI score compared with the HC group. Compared with the BPPV group, the VM group had significantly less ST (*p* = 0.00004) and lower SE (*p* = 0.004); compared with both the VM group and BPPV group, all the seven factors were significantly lower in the HC group (Figure 1).

According to the total PSQI score, there were 12 patients with good sleep quality, 14 patients with moderate sleep quality, and 31 patients with poor sleep quality in the BPPV group. Meanwhile, there were 5 patients with good sleep quality, 5 patients with moderate sleep quality, and 38 patients with poor sleep quality in the VM group. There were 28 patients with good sleep quality, 8 patients with moderate sleep quality, and 6 patients with poor sleep quality in the HC group. The results showed that the proportion of patients with sleep disorders was significantly higher in the VM group (79.16%) than in the BPPV group (54.39%) (*p* = 0.008) and was significantly higher in both the VM group (*p* < 0.00001) and BPPV group (*p* = 0.00004) than in the HC groups (14.28%).

If a factor of one patient was with grade > 1, then we defined this factor as an abnormal factor in this patient. We found 12 patients and 20 patients in the BPPV group and VM group, respectively, with abnormal sleep quality (*p* = 0.022). Meanwhile, there were 29 patients and 34 patients in the BPPV group and VM group, respectively, with abnormal SL (*p* = 0.038); there were 18 patients and 37 patients in the BPPV group and VM group, respectively, with abnormal ST (*p* < 0.001). The detailed information is described in Table 2. Compared with the HCs, the number of patients with abnormal factors in all the seven factors was significantly more in both the VM group and the BPPV group.

**Table 2:** Number of patients with abnormal factor in three groups.

**Factors**	**BPPV group**	**VM group**	**HCs group**	***P* ^**a**^**	***P* ^**b**^**	***P* ^**c**^**
Sleep quality	12 (21.05%)	20 (41.67%)	1 (2.4%)	0.022	0.007	<0.001
SL	29 (50.88%)	34 (70.83%)	4 (9.5%)	0.038	0.001	<0.001
ST	18 (31.58%)	37 (77.08%)	2 (4.8%)	<0.001	0.001	<0.001
SE	26 (45.61%)	34 (70.83%)	6 (14.3%)	0.133	0.001	<0.001
SDRF	21 (36.84%)	21 (43.75%)	1 (2.4%)	0.472	<0.001	<0.001
Hypnotics	11 (19.30%)	5 (10.42%)	0 (0%)	0.207	0.003	0.031
Daytime function	22 (38.60%)	18 (37.50%)	0 (0%)	0.908	<0.001	<0.001

a *P-value was obtained from BPPV group vs. VM group*.

b *P-value was obtained from BPPV group vs. HC group*.

c *P-value was obtained from VM group vs. HC group*.

*SL, sleep latency; ST, sleep time; SE, sleep efficiency; SDRF, sleep disorder-related factors; BPPV, benign paroxysmal positional vertigo; VM, vestibular migraine*.

### Objective Sleep Quality Evaluation

Compared with the HC group, we found that (i) the total ST was significantly lower in both the BPPV group (*p* = 0.002) and the VM group (*p* = 0.002), (ii) the SE was significantly lower in both the BPPV group (*p* = 0.003) and the VM group (*p* = 0.001), (iii) the WASO was significantly higher in both the BPPV group (*p* < 0.001) and the VM group (*p* < 0.001), (iv) the number of wake-ups was significantly higher in both the BPPV group (*p* < 0.001) and the VM group (*p* < 0.001), (v) the N1 (%) was significantly higher in both the BPPV group (*p* < 0.001) and the VM group (*p* < 0.001), (vi) the N2 (%) and N3 (%) was significantly higher and lower, respectively, in the VM group (*p* < 0.001 and *p* < 0.001, respectively), and (vii) the REM (%) was significantly lower in the VM group (*p* = 0.002). Meanwhile, we found that compared with the BPPV group, the VM group had significantly lower SE (*p* < 0.001), N3 (*p* < 0.001), and REM (%) (*p* = 0.008) and higher WASO (*p* < 0.001), N1 (*p* < 0.001), and N2 (*p* < 0.001). The detailed information is described in Table 3.

**Table 3:** Comparison of objective sleep quality evaluation indexes in three groups.

**Indexes**	**BPPV group**	**VM group**	**HCs group**	***P* ^**a**^**	***P* ^**b**^**	***P* ^**c**^**
Total ST (minute)	349.19 ± 66.72	331.95 ± 73.68	432.44 ± 37.89	0.211	0.002	0.002
SE (%)	69.70 ± 11.32	57.39 ± 10.25	80.01 ± 5.92	<0.001	0.003	0.001
SL (minute)	26.06 ± 19.12	28.09 ± 21.67	27.55 ± 12.38	0.611	0.25	0.48
REM-SL (minute)	118.50 ± 65.25	109.50 ± 79.54	108.13 ± 54.48	0.526	0.15	0.30
WASO (minute)	125.32 ± 53.44	199.64 ± 69.04	39.51 ± 24.96	<0.001	<0.001	<0.001
Number of wake-up	29 (25, 43)	35 (28, 46)	8 (5, 15)	0.431	<0.001	<0.001
N1 (%)	10.61 ± 5.49	15.25 ± 3.97	8.50 ± 3.66	<0.001	<0.001	<0.001
N2 (%)	55.26 ± 8.314	60.35 ± 4.61	52.35 ± 6.32	<0.001	0.23	<0.001
N3 (%)	15.20 ± 8.71	8.07 ± 4.23	16.17 ± 4.03	<0.001	0.51	<0.001
REM (%)	18.93 ± 5.64	16.34 ± 3.79	21.19 ± 4.02	0.008	0.37	0.002

a *P-value was obtained from BPPV group vs. VM group*.

b *P-value was obtained from BPPV group vs. HCs group*.

c *P-value was obtained from VM group vs. HC group*.

*SL, sleep latency; ST, sleep time; SE, sleep efficiency; REM, rapid eye movement; WASO, time of wake-up after sleep onset; BPPV, benign paroxysmal positional vertigo; VM, vestibular migraine*.

In this study, there were 19 patients with mild OSAHS, 12 patients with moderate OSAHS, and 1 patient with severe OSAHS in the BPPV group, there were 17 patients with mild OSAHS and 9 patients with severe OSAHS in the VM group, and there were 2 patients with mild OSAHS in the HC group. Here, the incidence rate of OSAHS was similar between the BBPV group and the VM group (*p* = 0.839) but significantly higher in both the BBPV group (*p* < 0.001) and the VM group (*p* < 0.001) than in the HC group. Meanwhile, we found that compared with the BPPV group, the VM group had a significantly lower incidence rate of moderate OSAHS (*p* = 0.001) and a significantly higher incidence rate of severe OSAHS (*p* = 0.003). In addition, the number of patients with PLMS was 3 in the BPPV group, 10 in the VM group, and 0 in the HCs group. The difference in the number of patients with PLMS between the BBPV group and the VM group was significant (*p* = 0.016).

## Discussion

At present, there is no accepted epidemiological data on the incidence rate of sleep disorders. Previous studies reported that the incidence rate of sleep disorders in people older than 18 years in China was 11.6–29.38% (18–20). Zeitlhofer et al. conducted an international survey of insomnia and found that approximately 30% of the general population had sleep disorders (21). Here, the proportion of patients with sleep disorders was significantly higher in both the BPPV group and the VM group than in the HC group. In this study, according to the PSQI score, 79.16% of VM patients, 54.39% of BPPV patients, and 14.28% HCs had sleep disorders. Meanwhile, the patients in both the BPPV group and the VM group had significantly lower total ST and SE and significantly higher WASO, number of wake-ups, and N1 (%) compared with the HC group. Therefore, clinicians should consider the sleep quality of VM patients and BPPV patients when making a treatment plan.

In this study, we found that the proportion of patients with sleep disorders was significantly higher in the VM group than in the BPPV group. The patients in the VM group had significantly lower SE, N3, and REM and significantly higher WASO, N1 (%), and N2 (%) compared with patients in the BPPV group. The PSQI score used here is the score index of sleep conditions in recent 1 month (22). However, according to the characteristics of BPPV, the occurrence of sleep disorders is longer than the onset time of BPPV. Many included BPPV patients reported that they experienced sleep disorders before the BPPV was diagnosed. Therefore, sleep disorders might be a risk factor for BPPV, but this hypothesis was needed in future studies to validate through exploring the sleep condition before the onset of BPPV.

Both VM and BPPV were recurrent and paroxysmal vertigo, but these two similar diseases had different pathogenesis (7, 8). In BPPV, the otolith abscission and displacement could produce mechanical stimulation on the semicircular ampullary ridge, which finally resulted in vertigo (23). However, in VM, some researchers believed that the disorder of the central nervous system (CNS) functions increased the excitability of trigeminal caudate nucleus, solitary tract nucleus, and vestibular nucleus, which finally resulted in vertigo (24), and some researchers assumed that the abnormal trigeminal neurovascular pathway caused the asymmetric release of neurotransmitters, such as serotonin and norepinephrine, on both sides of the vestibule, which finally resulted in vertigo (25). Thus, the different pathogenesis might be the cause of the different incidence rates of sleep disorders between VM and BPPV.

The proportion of patients with OSAHS was significantly higher in both the BPPV group and the VM group than in the HC group. OSAHS is a disease with a high incidence rate and great harm. Han et al. found that the positive rate of caloric tests in OSAHS patients was quite high (26), which indicated the high incidence rate of vestibular dysfunction in OSAHS patients. Patients with OSAHS might have intermittent hypoxia, which could result in abnormal dopamine metabolism. The vestibular nerve and parabrachial nucleus have a role in dopamine metabolism (27). These results suggested that there might be a close relationship between OSAHS and VM/BPPV, and clinicians should pay attention to the comorbidity of OSAHS in the diagnosis and treatment of patients with vertigo.

There were 10 VM patients who experienced PLMS in this study. Telles et al. reported that spinal cord injury might be a trigger to develop PLMS (28). The CNS is very important to our health (29–31); its lesions cause the spinal cord to lose the inhibitory effect of the pyramidal tract, which results in the occurrence of PLMS originating from the upper segment of the spinal cord. Some studies suggested that the production of PLMS was dominated by the pontine level and above the nervous system (32, 33). Almost all the theories about the pathogenesis of PLMS tend to think that it is caused by central abnormalities. The high incidence rate of PLMS might be related to the pathogenesis of VM. Although the pathogenesis of VM was still unclear, these results showed that the CNS might have an important role in the onset of VM.

## Limitations

Some shortcomings in this study should be mentioned here: (i) the number of included participants was relatively small, which made our findings needing future studies to validate further and support (34, 35); (ii) all of the included BPPV and VM patients were from the same place, which could limit the applicability of our results; (iii) we only explored the sleep disorders of two kinds of neuropsychiatric disorders; neurotologists should further investigate the sleep problems of patients with other neuropsychiatric disorders, such as depression (36–38); (iv) sex and age were two mainly confounding factors in life science researches (39–41). In this study, limited by the relatively small sample size, we did not analyze whether there were sex- or age-specific sleep disorders in patients with VM or BPPV; this point was worthy of further investigations.

## Conclusion

Our study found that the incidence rate of sleep disorders was significantly higher in both VM and BPPV patients than in the HC group. Meanwhile, compared with BPPV patients, the VM patients had the worse sleep quality, significantly lower sleep efficiency and N3, and significantly higher time of wake-up after sleep onset, N1, and N2. In addition, the VM patients had significantly higher incidence rates of severe OSAHS and PLMS. Our results indicated that to improve the curative effects, more attention should be paid to the comorbidity of sleep disorders in treating VM and BPPV.

## Data Availability Statement

The raw data supporting the conclusions of this article will be made available by the authors, without undue reservation.

## Ethics Statement

The studies involving human participants were reviewed and approved by Ethical Committee of Baotou Central Hospital. The patients/participants provided their written informed consent to participate in this study.

## Author Contributions

HX, BW, TM, and WX conceived and designed the study. HX, SZ, and QW performed the experiments. BW, XZ, and MK analyzed the data. HX, TM, and WX wrote the paper. All authors have read and approved the final version of this manuscript.

## Conflict of Interest

The authors declare that the research was conducted in the absence of any commercial or financial relationships that could be construed as a potential conflict of interest.

## Publisher's Note

All claims expressed in this article are solely those of the authors and do not necessarily represent those of their affiliated organizations, or those of the publisher, the editors and the reviewers. Any product that may be evaluated in this article, or claim that may be made by its manufacturer, is not guaranteed or endorsed by the publisher.
